# Crystal structure of *catena*-poly[[[tri­aqua­(4-cyano­benzoato-κ*O*)nickel(II)]-μ-4,4′-bi­pyridine-κ^2^ *N*:*N*′] 4-cyano­benzoate]

**DOI:** 10.1107/S2056989015018344

**Published:** 2015-10-17

**Authors:** Alfredo A. Morales-Tapia, Raúl Colorado-Peralta, Angélica M. Duarte-Hernández, Angelina Flores-Parra, José María Rivera

**Affiliations:** a Facultad de Ciencias Químicas, Universidad Veracruzana, Prolongación Oriente 6, No. 1009, Colonia Rafael Alvarado, CP 94340, Orizaba, Veracruz, Mexico; b Departamento de Química, Centro de Investigación y de Estudios Avanzados del Instituto Politécnico Nacional, CP 07360, México, D.F., Mexico

**Keywords:** crystal structure, nickel(II), 4-cyano­benzoate, 4,4′-bi­pyridine, polymeric complex salt, hydrogen bonding, π–π stacking

## Abstract

In the title polymeric complex salt, {[Ni(C_8_H_4_NO_2_)(C_10_H_8_N_2_)(H_2_O)_3_](C_8_H_4_NO_2_)}_*n*_, the Ni^II^ cation is coordinated by a 4-cyano­benzoate anion, two 4,4′-bi­pyridine ligands and three water mol­ecules in a distorted N_2_O_4_ octa­hedral geometry. The 4,4′-bi­pyridine ligands bridge the Ni^II^ cations to form polymeric chains of the title complex cations, propagating along the *c*-axis direction. The dihedral angle between the pyridine rings of the 4,4′-bi­pyridine ligand is 24.9 (6)°. In the crystal, the uncoordinating 4-cyano­benzoate anions link with the complex cations *via* O—H⋯O hydrogen bonds into a three-dimensional supra­molecular architecture. Weak C—H⋯O, C—H⋯N inter­actions and π–π stacking [centroid-to-centroid distances = 3.566 (4) and 3.885 (4) Å] are also observed in the crystal.

## Related literature   

For polymer structures reported with monodentate 4-cyano­benzoate and 4,4′-bipyridyl ligands coordinating to cobalt(II) and copper(II), see: He *et al.* (2003 ▸); He & Zhu (2003 ▸). For metal–organic structures with monodentate benzoato and 4,4′-bipyridyl ligands coordinating to nickel(II), see: Biradha *et al.* (1999 ▸); Song *et al.* (2009 ▸). For potential applications of the title compound, see: Peña-Rodríguez *et al.* (2014 ▸); Song *et al.* (2009 ▸).
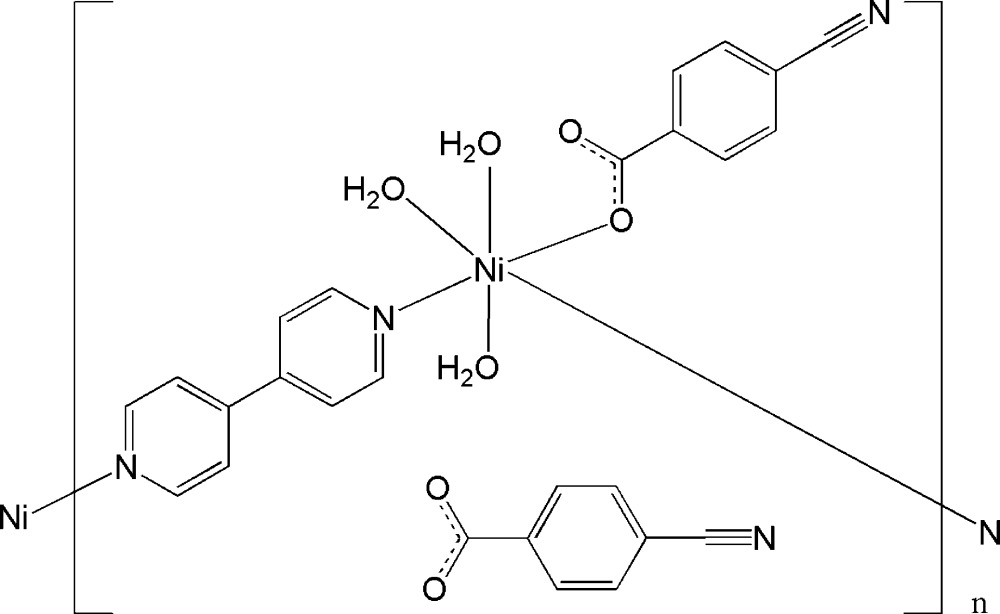


## Experimental   

### Crystal data  

[Ni(C_8_H_4_NO_2_)(C_10_H_8_N_2_)(H_2_O)_3_](C_8_H_4_NO_2_)
*M* *_r_* = 561.19
Monoclinic, 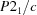

*a* = 7.176 (5) Å
*b* = 21.373 (9) Å
*c* = 17.032 (9) Å
β = 110.32 (3)°
*V* = 2450 (2) Å^3^
*Z* = 4
Mo *K*α radiation
μ = 0.85 mm^−1^
*T* = 293 K
0.10 × 0.05 × 0.05 mm

### Data collection  

Nonius KappaCCD diffractometer
Absorption correction: multi-scan (North *et al.*, 1968 ▸) *T*_min_ = 0.872, *T*_max_ = 0.969
19002 measured reflections
5632 independent reflections
2419 reflections with *I* > 2σ(*I*)
*R*_int_ = 0.143

### Refinement  

*R*[*F* ^2^ > 2σ(*F* ^2^)] = 0.062
*wR*(*F* ^2^) = 0.126
*S* = 0.97
5632 reflections
367 parameters
6 restraints
H atoms treated by a mixture of independent and constrained refinement
Δρ_max_ = 0.38 e Å^−3^
Δρ_min_ = −0.38 e Å^−3^

### 

Data collection: *COLLECT* (Bruker, 2004 ▸); cell refinement: *SCALEPACK* (Otwinowski & Minor, 1997 ▸); data reduction: *DENZO* (Otwinowski & Minor, 1997 ▸) and *SCALEPACK*; program(s) used to solve structure: *SHELXS97* (Sheldrick, 2008 ▸); program(s) used to refine structure: *SHELXL97* (Sheldrick, 2008 ▸); molecular graphics: *Mercury* (Macrae *et al.*, 2006 ▸); software used to prepare material for publication: *WinGX* (Farrugia, 2012 ▸), *enCIFer* (Allen *et al.*, 2004 ▸) and *publCIF* (Westrip, 2010 ▸).

## Supplementary Material

Crystal structure: contains datablock(s) global, I. DOI: 10.1107/S2056989015018344/xu5875sup1.cif

Structure factors: contains datablock(s) I. DOI: 10.1107/S2056989015018344/xu5875Isup2.hkl

**Fig. 1.**
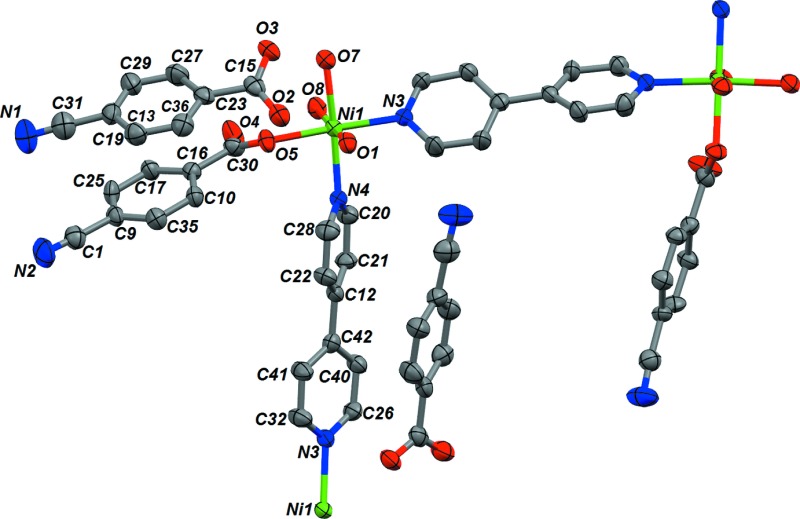
The molecular structure of the title compound, showing the atom-labelling scheme. Displacement ellipsoids are drawn at the 50% probability level, H atoms are omitted for clarity.

. DOI: 10.1107/S2056989015018344/xu5875fig1.tif
The mol­ecular structure of the title compound, showing the atom-labelling scheme. Displacement ellipsoids are drawn at the 50% probability level, H atoms are omitted for clarity.

CCDC reference: 1428986

Additional supporting information:  crystallographic information; 3D view; checkCIF report

## Figures and Tables

**Table 1:** Hydrogen-bond geometry (, )

*D*H*A*	*D*H	H*A*	*D* *A*	*D*H*A*
O1H1*A*O3^i^	0.85(1)	1.88(1)	2.715(5)	167(4)
O1H1*B*O2	0.84(4)	2.09(4)	2.882(5)	156(4)
O7H7*A*O2^i^	0.84(2)	1.94(2)	2.777(5)	172(4)
O7H7*B*O3	0.83(7)	1.97(7)	2.761(5)	157(8)
O8H8*A*O2^ii^	0.85(5)	2.07(5)	2.901(5)	165(6)
O8H8*B*O4	0.84(4)	1.81(5)	2.619(5)	162(7)
C32H32N1^iii^	0.93	2.43	3.121(8)	131
C35H35O4^iv^	0.93	2.42	3.234(7)	146

Symmetry codes: (i) 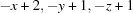; (ii) 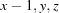; (iii) 
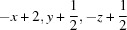
; (iv) 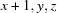.

## supplementary crystallographic information

### S1. Comment

The design of metal-organic frameworks is of current interest in the fields of supramolecular chemistry and crystal engineering. This interest stems from their potential applications as functional materials, such as in gas storage, ion-exchange, catalysis, magnetism and molecular sensing (Peña-Rodríguez *et al.*, 2014; Song *et al.* 2009). In the field of crystal engineering, 4,4'-bipyridine has been extensively used to construct novel one-, two-, and three dimensional coordination polymers with potential applications as functional materials. The combination of 4,4'-bipyridine and carboxylic acid is largely directed toward interesting topologies (Biradha *et al.* 1999). 4-cyanobenzoic acid has been used to develop fluorescent materials (He & Zhu 2003*a,b*).

4,4'-Bipyridine is an excellent, rigid bridging ligand for the construction of novel metal-organic frameworks due to its various coordinative modes with metal ions. Currently all the metal-organic coordination compounds obtained with cyanobenzoic acid and 4,4'-bipyridine contain the cyanobenzoato group as mono- or bidentate ligand, the title compound is the first example of a polymeric structure with cyanobenzoate as a counter ion.

The title compound is a nickel(II) polymeric complex cation (Fig. 1) together with four cyanobenzoate counter ions in the unit cell. Each nickel(II) ion displays a distorted octahedral coordination geometry being surrounded by three *O*-donor molecules of water, one *O*-donor molecule of 4-cyanobenzoato and two *N*-donor molecules *trans*-disposed of 4,4'-bipyridyl. The dihedral angle between the aromatic rings of the 4,4'-bipyridine ligand is 24.9 (6)° (ligand containing N3 and N4).

In the crystal, the uncoordinate 4-cyanobenzoate anions link with the complex cations via O—H···O hydrogen bonds into the three dimensional supramolecular architecture. Weak C—H···O, C—H···N and π-π stacking [centroid-to-centroid distances = 3.566 (4) and 3.885 (4) Å] are also observed in the crystal.

### S2. Experimental

A solution of nickel(II) nitrate hexahydrate (62.1 mg, 0.21 mmol) in 5 mL of deionized water was added dropwise to 5 mL of a methanol solution of 4,4'-bipyridine (50 mg, 0.32 mmol), the reaction mixture was refluxed for two hours; after which a solution of 4-cyanobenzoic acid (62.8 mg, 0.42 mmol) in 5 mL of DMF was slowly added at room temperature, the reaction mixture was refluxed for five hours. The solid was crystallized from the solution giving blue crystals of the title compound which were suitable for X-ray crystal structure analysis and fully characterized by standard analytical methods. *M.p.* > 350°C.

### S3. Refinement

The water H atoms were located in a difference Fourier map and refined with a distance restraint O—H = 0.84 Å, *U*_iso_(H) = 1.2*U*_eq_(O). Other H atoms were positioned geometrically and refined using a riding model approximation with distance C—H = 0.93 Å, *U*_iso_(H) = 1.2*U*_eq_(C).

### Crystal data

**Table:**

[Ni(C_8_H_4_NO_2_)(C_10_H_8_N_2_)(H_2_O)_3_](C_8_H_4_NO_2_)	*F*(000) = 1160
*M**_r_* = 561.19	*D*_x_ = 1.521 Mg m^−^^3^
Monoclinic, *P*2_1_/*c*	Melting point: 350 K
Hall symbol: -P 2ybc	Mo *K*α radiation, λ = 0.71073 Å
*a* = 7.176 (5) Å	Cell parameters from 10938 reflections
*b* = 21.373 (9) Å	θ = 2.9–27.5°
*c* = 17.032 (9) Å	µ = 0.85 mm^−^^1^
β = 110.32 (3)°	*T* = 293 K
*V* = 2450 (2) Å^3^	Needle, blue
*Z* = 4	0.1 × 0.05 × 0.05 mm

### Data collection

**Table:**

Nonius KappaCCD diffractometer	5632 independent reflections
Radiation source: Enraf Nonius FR590	2419 reflections with *I* > 2σ(*I*)
Graphite monochromator	*R*_int_ = 0.143
Detector resolution: 9 pixels mm^-1^	θ_max_ = 27.6°, θ_min_ = 3.2°
CCD rotation images, thick slices scans	*h* = −9→9
Absorption correction: multi-scan (North *et al.*, 1968)	*k* = −27→24
*T*_min_ = 0.872, *T*_max_ = 0.969	*l* = −22→18
19002 measured reflections	

### Refinement

**Table:**

Refinement on *F*^2^	Primary atom site location: structure-invariant direct methods
Least-squares matrix: full	Secondary atom site location: difference Fourier map
*R*[*F*^2^ > 2σ(*F*^2^)] = 0.062	Hydrogen site location: inferred from neighbouring sites
*wR*(*F*^2^) = 0.126	H atoms treated by a mixture of independent and constrained refinement
*S* = 0.97	*w* = 1/[σ^2^(*F*_o_^2^) + (0.0333*P*)^2^] where *P* = (*F*_o_^2^ + 2*F*_c_^2^)/3
5632 reflections	(Δ/σ)_max_ < 0.001
367 parameters	Δρ_max_ = 0.38 e Å^−^^3^
6 restraints	Δρ_min_ = −0.38 e Å^−^^3^

### Special details

**Table:**

Geometry. All e.s.d.'s (except the e.s.d. in the dihedral angle between two l.s. planes) are estimated using the full covariance matrix. The cell e.s.d.'s are taken into account individually in the estimation of e.s.d.'s in distances, angles and torsion angles; correlations between e.s.d.'s in cell parameters are only used when they are defined by crystal symmetry. An approximate (isotropic) treatment of cell e.s.d.'s is used for estimating e.s.d.'s involving l.s. planes.
Refinement. Refinement of *F*^2^ against ALL reflections. The weighted *R*-factor *wR* and goodness of fit *S* are based on *F*^2^, conventional *R*-factors *R* are based on *F*, with *F* set to zero for negative *F*^2^. The threshold expression of *F*^2^ > σ(*F*^2^) is used only for calculating *R*-factors(gt) *etc*. and is not relevant to the choice of reflections for refinement. *R*-factors based on *F*^2^ are statistically about twice as large as those based on *F*, and *R*-factors based on ALL data will be even larger.

### Fractional atomic coordinates and isotropic or equivalent isotropic displacement parameters (Å^2^)

**Table:**

	*x*	*y*	*z*	*U*_iso_*/*U*_eq_	
C1	0.8802 (7)	0.3998 (2)	−0.0400 (3)	0.0443 (13)	
C9	0.7907 (7)	0.42600 (19)	0.0174 (3)	0.0360 (11)	
C10	0.8269 (7)	0.48622 (18)	0.1392 (3)	0.0353 (12)	
H10	0.9042	0.5118	0.1824	0.042*	
C12	0.5875 (7)	0.73382 (18)	0.1336 (3)	0.0317 (11)	
C16	0.6335 (7)	0.47103 (18)	0.1338 (3)	0.0318 (11)	
C17	0.5189 (7)	0.43439 (19)	0.0674 (3)	0.0386 (12)	
H17	0.3888	0.4248	0.0623	0.046*	
C20	0.4377 (7)	0.68161 (19)	0.2211 (3)	0.0400 (12)	
H20	0.3264	0.6758	0.2363	0.048*	
C21	0.4258 (7)	0.72438 (18)	0.1591 (3)	0.0362 (11)	
H21	0.3094	0.747	0.1343	0.043*	
C22	0.7544 (7)	0.69861 (19)	0.1740 (3)	0.0393 (12)	
H22	0.8665	0.703	0.159	0.047*	
C25	0.5952 (7)	0.41170 (19)	0.0083 (3)	0.0407 (12)	
H25	0.5172	0.3875	−0.0365	0.049*	
C26	0.4655 (7)	0.63031 (18)	0.4810 (3)	0.0367 (12)	
H26	0.3831	0.5954	0.4691	0.044*	
C28	0.7576 (7)	0.65723 (19)	0.2359 (3)	0.0411 (12)	
H28	0.8733	0.6346	0.2621	0.049*	
C30	0.5515 (8)	0.49599 (19)	0.1980 (3)	0.0346 (11)	
C32	0.6965 (8)	0.6908 (2)	0.4545 (3)	0.0463 (14)	
H32	0.779	0.6986	0.4239	0.056*	
C35	0.9040 (7)	0.46355 (19)	0.0811 (3)	0.0387 (12)	
H35	1.033	0.4738	0.0852	0.046*	
C40	0.4596 (7)	0.67016 (18)	0.5451 (3)	0.0369 (12)	
H40	0.3754	0.6615	0.5747	0.044*	
C41	0.6984 (8)	0.7328 (2)	0.5164 (3)	0.0472 (14)	
H41	0.7795	0.768	0.5261	0.057*	
C42	0.5795 (7)	0.77749 (18)	0.0643 (3)	0.0333 (11)	
N2	0.9595 (7)	0.3780 (2)	−0.0811 (3)	0.0675 (14)	
N3	0.5819 (6)	0.63939 (14)	0.4361 (2)	0.0325 (9)	
N4	0.6007 (6)	0.64792 (14)	0.2606 (2)	0.0309 (9)	
O1	0.9251 (5)	0.58567 (15)	0.40016 (19)	0.0365 (8)	
O4	0.3679 (6)	0.49516 (16)	0.1789 (2)	0.0592 (10)	
O5	0.6741 (4)	0.51639 (12)	0.26558 (18)	0.0354 (8)	
O7	0.6401 (5)	0.50203 (15)	0.4306 (2)	0.0378 (8)	
O8	0.3102 (5)	0.55901 (15)	0.2997 (2)	0.0384 (8)	
Ni1	0.61430 (9)	0.57627 (2)	0.34785 (3)	0.02972 (18)	
C13	0.9693 (8)	0.3155 (2)	0.1751 (3)	0.0431 (13)	
C15	1.0418 (7)	0.4313 (2)	0.3870 (3)	0.0391 (12)	
C19	1.1431 (8)	0.3493 (2)	0.2086 (3)	0.0467 (13)	
H19	1.2423	0.3469	0.1853	0.056*	
C23	1.0206 (8)	0.3895 (2)	0.3140 (3)	0.0392 (12)	
C27	0.8511 (8)	0.3536 (2)	0.2800 (3)	0.0495 (14)	
H27	0.7541	0.3541	0.3046	0.059*	
C29	0.8237 (8)	0.3171 (2)	0.2105 (3)	0.0530 (14)	
H29	0.708	0.2938	0.1876	0.064*	
C31	0.9341 (8)	0.2797 (2)	0.0993 (3)	0.0526 (14)	
C36	1.1666 (7)	0.3871 (2)	0.2782 (3)	0.0431 (12)	
H36	1.2813	0.411	0.3007	0.052*	
N1	0.9046 (7)	0.2533 (2)	0.0379 (3)	0.0686 (14)	
O2	1.1613 (5)	0.47669 (14)	0.39992 (19)	0.0476 (9)	
O3	0.9334 (5)	0.41867 (13)	0.42993 (18)	0.0440 (8)	
H1A	0.963 (6)	0.5900 (19)	0.4528 (7)	0.048 (15)*	
H1B	0.974 (7)	0.5543 (14)	0.385 (3)	0.065 (18)*	
H7A	0.691 (6)	0.5104 (19)	0.4819 (9)	0.048 (15)*	
H7B	0.716 (9)	0.479 (3)	0.416 (5)	0.16 (3)*	
H8A	0.287 (9)	0.536 (2)	0.336 (3)	0.10 (2)*	
H8B	0.303 (10)	0.537 (2)	0.258 (2)	0.11 (3)*	

### Atomic displacement parameters (Å^2^)

**Table:**

	*U*^11^	*U*^22^	*U*^33^	*U*^12^	*U*^13^	*U*^23^
C1	0.045 (4)	0.045 (3)	0.040 (3)	0.005 (2)	0.012 (3)	−0.001 (2)
C9	0.044 (3)	0.035 (2)	0.033 (3)	0.007 (2)	0.018 (2)	−0.003 (2)
C10	0.039 (3)	0.036 (3)	0.029 (3)	−0.002 (2)	0.009 (3)	−0.006 (2)
C12	0.039 (3)	0.033 (2)	0.025 (3)	−0.004 (2)	0.014 (2)	−0.0022 (19)
C16	0.034 (3)	0.030 (2)	0.030 (3)	−0.001 (2)	0.010 (2)	−0.0001 (19)
C17	0.039 (3)	0.045 (3)	0.034 (3)	−0.004 (2)	0.015 (2)	−0.006 (2)
C20	0.038 (3)	0.044 (3)	0.041 (3)	0.005 (2)	0.018 (3)	0.007 (2)
C21	0.043 (3)	0.034 (2)	0.034 (3)	0.004 (2)	0.016 (3)	0.010 (2)
C22	0.041 (3)	0.047 (3)	0.035 (3)	0.003 (2)	0.020 (3)	0.007 (2)
C25	0.046 (4)	0.039 (3)	0.034 (3)	−0.004 (2)	0.010 (3)	−0.013 (2)
C26	0.044 (3)	0.030 (2)	0.040 (3)	−0.009 (2)	0.019 (3)	−0.004 (2)
C28	0.038 (3)	0.045 (3)	0.039 (3)	0.006 (2)	0.012 (3)	0.008 (2)
C30	0.032 (3)	0.040 (3)	0.034 (3)	−0.002 (2)	0.015 (3)	0.000 (2)
C32	0.059 (4)	0.046 (3)	0.046 (3)	−0.019 (3)	0.034 (3)	−0.014 (2)
C35	0.039 (3)	0.043 (3)	0.034 (3)	−0.001 (2)	0.013 (3)	−0.004 (2)
C40	0.041 (3)	0.043 (3)	0.031 (3)	−0.006 (2)	0.019 (3)	−0.004 (2)
C41	0.066 (4)	0.040 (3)	0.045 (3)	−0.021 (2)	0.032 (3)	−0.013 (2)
C42	0.036 (3)	0.030 (2)	0.034 (3)	−0.003 (2)	0.011 (2)	−0.003 (2)
N2	0.075 (4)	0.069 (3)	0.078 (4)	−0.002 (3)	0.050 (3)	−0.022 (3)
N3	0.043 (3)	0.030 (2)	0.028 (2)	−0.0065 (18)	0.017 (2)	−0.0009 (16)
N4	0.038 (3)	0.029 (2)	0.028 (2)	−0.0038 (18)	0.014 (2)	−0.0007 (16)
O1	0.039 (2)	0.042 (2)	0.026 (2)	−0.0005 (16)	0.0079 (17)	−0.0057 (15)
O4	0.042 (3)	0.095 (3)	0.045 (2)	−0.013 (2)	0.020 (2)	−0.0303 (19)
O5	0.036 (2)	0.0409 (17)	0.0245 (18)	0.0034 (14)	0.0047 (16)	−0.0058 (14)
O7	0.048 (2)	0.0384 (19)	0.028 (2)	−0.0040 (17)	0.0141 (19)	0.0000 (15)
O8	0.038 (2)	0.047 (2)	0.033 (2)	−0.0018 (16)	0.0160 (18)	−0.0056 (17)
Ni1	0.0363 (4)	0.0297 (3)	0.0249 (3)	−0.0020 (3)	0.0128 (3)	−0.0014 (3)
C13	0.057 (4)	0.035 (3)	0.039 (3)	0.005 (3)	0.018 (3)	0.000 (2)
C15	0.041 (3)	0.047 (3)	0.028 (3)	0.012 (3)	0.010 (2)	0.007 (2)
C19	0.047 (4)	0.049 (3)	0.050 (3)	0.005 (3)	0.025 (3)	0.000 (3)
C23	0.050 (4)	0.041 (3)	0.027 (3)	0.006 (3)	0.014 (3)	0.006 (2)
C27	0.057 (4)	0.055 (3)	0.043 (3)	−0.011 (3)	0.025 (3)	−0.005 (3)
C29	0.064 (4)	0.050 (3)	0.044 (3)	−0.011 (3)	0.017 (3)	−0.007 (2)
C31	0.045 (4)	0.057 (3)	0.050 (4)	0.009 (3)	0.009 (3)	−0.004 (3)
C36	0.040 (4)	0.044 (3)	0.040 (3)	0.004 (2)	0.007 (3)	0.000 (2)
N1	0.060 (4)	0.080 (3)	0.064 (3)	0.006 (3)	0.019 (3)	−0.027 (3)
O2	0.057 (3)	0.051 (2)	0.039 (2)	−0.0047 (18)	0.0220 (19)	−0.0062 (16)
O3	0.051 (2)	0.0528 (19)	0.0325 (18)	0.0030 (17)	0.0202 (17)	0.0022 (15)

### Geometric parameters (Å, º)

**Table:**

C1—N2	1.144 (5)	C40—H40	0.93
C1—C9	1.455 (6)	C41—C42^i^	1.388 (6)
C9—C35	1.367 (6)	C41—H41	0.93
C9—C25	1.391 (6)	C42—C40^ii^	1.379 (5)
C10—C35	1.379 (5)	C42—C41^ii^	1.388 (6)
C10—C16	1.397 (6)	N3—Ni1	2.092 (3)
C10—H10	0.93	N4—Ni1	2.113 (3)
C12—C22	1.379 (6)	O1—Ni1	2.104 (3)
C12—C21	1.388 (5)	O1—H1A	0.846 (10)
C12—C42	1.490 (5)	O1—H1B	0.840 (10)
C16—C17	1.387 (6)	O5—Ni1	2.050 (3)
C16—C30	1.507 (6)	O7—Ni1	2.088 (3)
C17—C25	1.390 (5)	O7—H7A	0.840 (10)
C17—H17	0.93	O7—H7B	0.838 (10)
C20—N4	1.339 (5)	O8—Ni1	2.080 (3)
C20—C21	1.376 (5)	O8—H8A	0.842 (10)
C20—H20	0.93	O8—H8B	0.839 (10)
C21—H21	0.93	C13—C29	1.376 (6)
C22—C28	1.370 (5)	C13—C19	1.381 (6)
C22—H22	0.93	C13—C31	1.445 (7)
C25—H25	0.93	C15—O2	1.262 (5)
C26—N3	1.329 (5)	C15—O3	1.267 (5)
C26—C40	1.397 (5)	C15—C23	1.496 (6)
C26—H26	0.93	C19—C36	1.396 (6)
C28—N4	1.346 (5)	C19—H19	0.93
C28—H28	0.93	C23—C27	1.384 (6)
C30—O4	1.243 (5)	C23—C36	1.384 (6)
C30—O5	1.259 (5)	C27—C29	1.373 (6)
C32—N3	1.343 (5)	C27—H27	0.93
C32—C41	1.382 (6)	C29—H29	0.93
C32—H32	0.93	C31—N1	1.141 (6)
C35—H35	0.93	C36—H36	0.93
C40—C42^i^	1.379 (6)		
			
N2—C1—C9	176.0 (5)	C26—N3—Ni1	124.6 (3)
C35—C9—C25	121.1 (4)	C32—N3—Ni1	118.9 (3)
C35—C9—C1	118.6 (4)	C20—N4—C28	116.3 (4)
C25—C9—C1	120.3 (4)	C20—N4—Ni1	124.2 (3)
C35—C10—C16	120.5 (4)	C28—N4—Ni1	119.2 (3)
C35—C10—H10	119.7	Ni1—O1—H1A	111 (3)
C16—C10—H10	119.7	Ni1—O1—H1B	107 (4)
C22—C12—C21	116.1 (4)	H1A—O1—H1B	114 (4)
C22—C12—C42	121.7 (4)	C30—O5—Ni1	126.5 (3)
C21—C12—C42	122.2 (4)	Ni1—O7—H7A	116 (3)
C17—C16—C10	118.6 (4)	Ni1—O7—H7B	99 (5)
C17—C16—C30	121.4 (4)	H7A—O7—H7B	110 (6)
C10—C16—C30	119.9 (4)	Ni1—O8—H8A	105 (4)
C16—C17—C25	121.1 (4)	Ni1—O8—H8B	100 (5)
C16—C17—H17	119.4	H8A—O8—H8B	108 (5)
C25—C17—H17	119.4	O5—Ni1—O8	93.42 (12)
N4—C20—C21	123.6 (4)	O5—Ni1—O7	89.81 (12)
N4—C20—H20	118.2	O8—Ni1—O7	88.07 (13)
C21—C20—H20	118.2	O5—Ni1—N3	174.58 (14)
C20—C21—C12	120.1 (4)	O8—Ni1—N3	91.99 (14)
C20—C21—H21	120	O7—Ni1—N3	90.62 (13)
C12—C21—H21	120	O5—Ni1—O1	84.62 (12)
C28—C22—C12	121.1 (4)	O8—Ni1—O1	175.05 (13)
C28—C22—H22	119.5	O7—Ni1—O1	87.38 (13)
C12—C22—H22	119.5	N3—Ni1—O1	90.01 (14)
C17—C25—C9	118.5 (4)	O5—Ni1—N4	86.63 (11)
C17—C25—H25	120.7	O8—Ni1—N4	93.74 (14)
C9—C25—H25	120.7	O7—Ni1—N4	176.10 (14)
N3—C26—C40	123.8 (4)	N3—Ni1—N4	92.78 (12)
N3—C26—H26	118.1	O1—Ni1—N4	90.69 (13)
C40—C26—H26	118.1	C29—C13—C19	121.3 (4)
N4—C28—C22	122.9 (4)	C29—C13—C31	118.7 (5)
N4—C28—H28	118.6	C19—C13—C31	119.9 (5)
C22—C28—H28	118.6	O2—C15—O3	125.4 (4)
O4—C30—O5	125.8 (4)	O2—C15—C23	118.1 (4)
O4—C30—C16	116.8 (4)	O3—C15—C23	116.5 (4)
O5—C30—C16	117.4 (4)	C13—C19—C36	118.6 (4)
N3—C32—C41	123.6 (4)	C13—C19—H19	120.7
N3—C32—H32	118.2	C36—C19—H19	120.7
C41—C32—H32	118.2	C27—C23—C36	119.0 (4)
C9—C35—C10	120.0 (4)	C27—C23—C15	120.0 (4)
C9—C35—H35	120	C36—C23—C15	120.9 (4)
C10—C35—H35	120	C29—C27—C23	121.2 (5)
C42^i^—C40—C26	119.6 (4)	C29—C27—H27	119.4
C42^i^—C40—H40	120.2	C23—C27—H27	119.4
C26—C40—H40	120.2	C27—C29—C13	119.2 (5)
C32—C41—C42^i^	120.0 (4)	C27—C29—H29	120.4
C32—C41—H41	120	C13—C29—H29	120.4
C42^i^—C41—H41	120	N1—C31—C13	177.6 (6)
C40^ii^—C42—C41^ii^	116.7 (4)	C23—C36—C19	120.6 (5)
C40^ii^—C42—C12	123.0 (4)	C23—C36—H36	119.7
C41^ii^—C42—C12	120.2 (4)	C19—C36—H36	119.7
C26—N3—C32	116.2 (4)		
			
N2—C1—C9—C35	52 (8)	C30—O5—Ni1—N3	−159.1 (12)
N2—C1—C9—C25	−127 (8)	C30—O5—Ni1—O1	−166.3 (3)
C35—C10—C16—C17	−2.0 (6)	C30—O5—Ni1—N4	−75.3 (3)
C35—C10—C16—C30	179.8 (4)	C26—N3—Ni1—O5	−139.7 (12)
C10—C16—C17—C25	1.6 (6)	C32—N3—Ni1—O5	34.9 (14)
C30—C16—C17—C25	179.8 (4)	C26—N3—Ni1—O8	42.9 (4)
N4—C20—C21—C12	0.9 (7)	C32—N3—Ni1—O8	−142.5 (4)
C22—C12—C21—C20	−0.3 (6)	C26—N3—Ni1—O7	−45.1 (4)
C42—C12—C21—C20	177.3 (4)	C32—N3—Ni1—O7	129.4 (4)
C21—C12—C22—C28	−0.4 (6)	C26—N3—Ni1—O1	−132.5 (4)
C42—C12—C22—C28	−178.0 (4)	C32—N3—Ni1—O1	42.0 (4)
C16—C17—C25—C9	0.6 (7)	C26—N3—Ni1—N4	136.8 (4)
C35—C9—C25—C17	−2.6 (7)	C32—N3—Ni1—N4	−48.6 (4)
C1—C9—C25—C17	176.0 (4)	C20—N4—Ni1—O5	117.3 (3)
C12—C22—C28—N4	0.6 (7)	C28—N4—Ni1—O5	−55.9 (3)
C17—C16—C30—O4	−16.5 (6)	C20—N4—Ni1—O8	24.1 (3)
C10—C16—C30—O4	161.7 (4)	C28—N4—Ni1—O8	−149.1 (3)
C17—C16—C30—O5	164.3 (4)	C20—N4—Ni1—O7	142 (2)
C10—C16—C30—O5	−17.6 (6)	C28—N4—Ni1—O7	−32 (2)
C25—C9—C35—C10	2.2 (7)	C20—N4—Ni1—N3	−68.1 (4)
C1—C9—C35—C10	−176.4 (4)	C28—N4—Ni1—N3	118.7 (3)
C16—C10—C35—C9	0.1 (6)	C20—N4—Ni1—O1	−158.1 (3)
N3—C26—C40—C42^i^	0.2 (7)	C28—N4—Ni1—O1	28.7 (3)
N3—C32—C41—C42^i^	0.9 (8)	C29—C13—C19—C36	−2.0 (7)
C22—C12—C42—C40^ii^	−153.1 (4)	C31—C13—C19—C36	175.3 (4)
C21—C12—C42—C40^ii^	29.4 (6)	O2—C15—C23—C27	−158.1 (4)
C22—C12—C42—C41^ii^	25.7 (6)	O3—C15—C23—C27	20.4 (6)
C21—C12—C42—C41^ii^	−151.8 (4)	O2—C15—C23—C36	20.3 (6)
C40—C26—N3—C32	−0.9 (7)	O3—C15—C23—C36	−161.2 (4)
C40—C26—N3—Ni1	173.8 (3)	C36—C23—C27—C29	−1.7 (7)
C41—C32—N3—C26	0.4 (7)	C15—C23—C27—C29	176.8 (4)
C41—C32—N3—Ni1	−174.6 (4)	C23—C27—C29—C13	1.2 (8)
C21—C20—N4—C28	−0.7 (6)	C19—C13—C29—C27	0.6 (7)
C21—C20—N4—Ni1	−174.2 (3)	C31—C13—C29—C27	−176.7 (5)
C22—C28—N4—C20	0.0 (6)	C29—C13—C31—N1	100 (14)
C22—C28—N4—Ni1	173.8 (3)	C19—C13—C31—N1	−77 (14)
O4—C30—O5—Ni1	−19.4 (6)	C27—C23—C36—C19	0.3 (7)
C16—C30—O5—Ni1	159.8 (3)	C15—C23—C36—C19	−178.2 (4)
C30—O5—Ni1—O8	18.3 (3)	C13—C19—C36—C23	1.5 (7)
C30—O5—Ni1—O7	106.3 (3)		

Symmetry codes: (i) *x*, −*y*+3/2, *z*+1/2; (ii) *x*, −*y*+3/2, *z*−1/2.

### Hydrogen-bond geometry (Å, º)

**Table:**

*D*—H···*A*	*D*—H	H···*A*	*D*···*A*	*D*—H···*A*
O1—H1*A*···O3^iii^	0.85 (1)	1.88 (1)	2.715 (5)	167 (4)
O1—H1*B*···O2	0.84 (4)	2.09 (4)	2.882 (5)	156 (4)
O7—H7*A*···O2^iii^	0.84 (2)	1.94 (2)	2.777 (5)	172 (4)
O7—H7*B*···O3	0.83 (7)	1.97 (7)	2.761 (5)	157 (8)
O8—H8*A*···O2^iv^	0.85 (5)	2.07 (5)	2.901 (5)	165 (6)
O8—H8*B*···O4	0.84 (4)	1.81 (5)	2.619 (5)	162 (7)
C32—H32···N1^v^	0.93	2.43	3.121 (8)	131
C35—H35···O4^vi^	0.93	2.42	3.234 (7)	146

Symmetry codes: (iii) −*x*+2, −*y*+1, −*z*+1; (iv) *x*−1, *y*, *z*; (v) −*x*+2, *y*+1/2, −*z*+1/2; (vi) *x*+1, *y*, *z*.
