# Supplementary figures and images for: Crystal structure of catena-poly[[[tri­aqua­(4-cyano­benzoato-κO)nickel(II)]-μ-4,4′-bi­pyridine-κ2 N:N′] 4-cyano­benzoate]
Source: Acta Crystallogr E Crystallogr Commun. 2015 Oct 17;71(Pt 11):m197–8. doi: 10.1107/S2056989015018344 (PMC4645088; doi:10.1107/S2056989015018344)

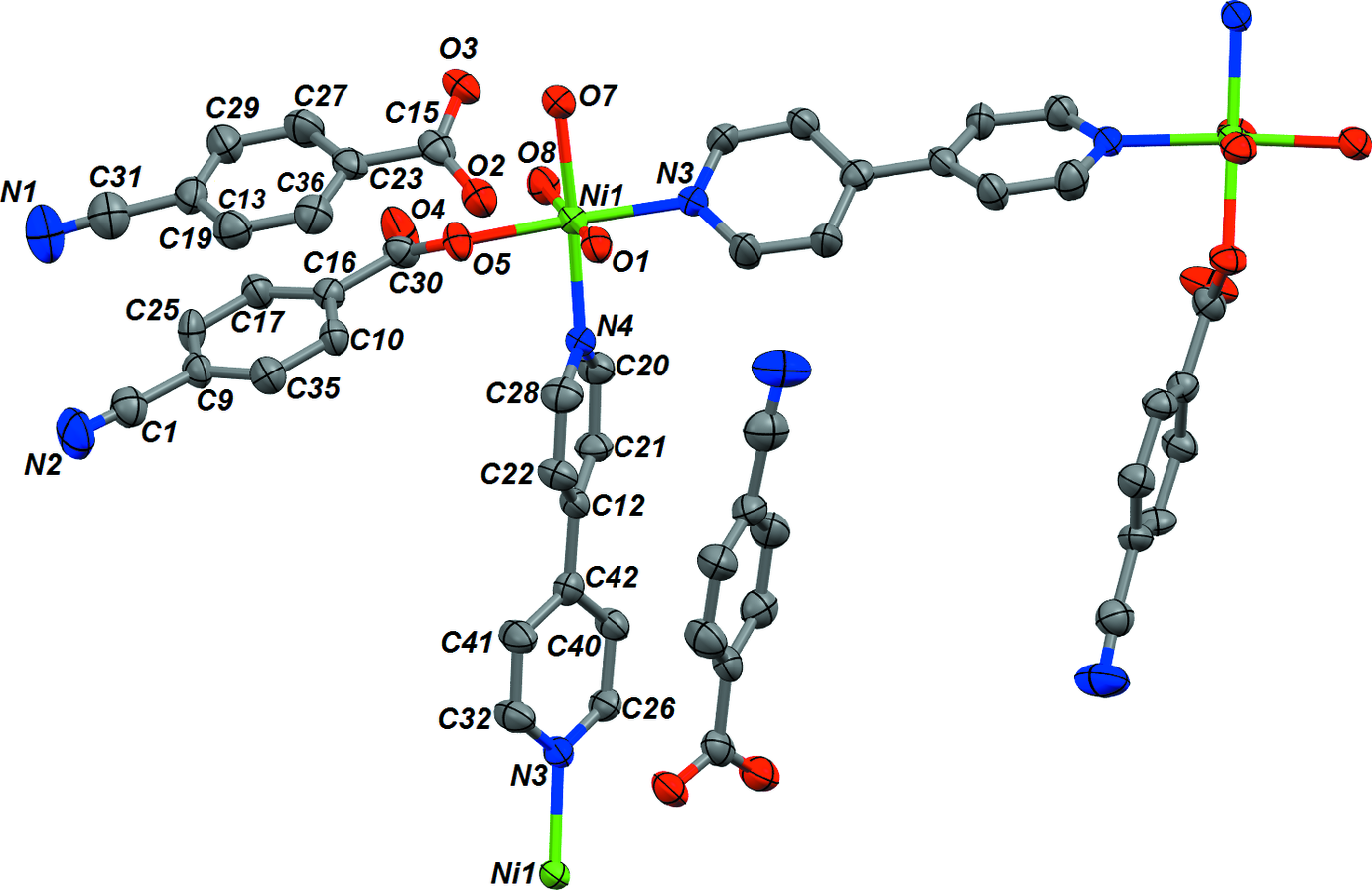

Supplement: Supplementary file 3 [file e-71-0m197-fig1.tif]
